# Genetic heterogeneity of residual variance for growth traits in American angus Cattle

**DOI:** 10.1093/jas/skag167

**Published:** 2026-05-23

**Authors:** Sabrina T Amorim, Kelli J Retallick, André Garcia, Noelia Ibañez-Escriche, Gota Morota

**Affiliations:** School of Animal Sciences, Virginia Tech, Blacksburg, VA 24061, United States; Department of Animal and Food Sciences, Oklahoma State University, Stillwater, OK 74078, United States; Angus Genetics Inc., American Angus Association, Saint Joseph, MO 64506, United States; Angus Genetics Inc., American Angus Association, Saint Joseph, MO 64506, United States; Institute for Animal Science and Technology, Universitat Politècnica de València, València 46022, Spain; School of Animal Sciences, Virginia Tech, Blacksburg, VA 24061, United States; Laboratory of Biometry and Bioinformatics, Department of Agricultural and Environmental Biology, Graduate School of Agricultural and Life Sciences, The University of Tokyo, Bunkyo, Tokyo 113-8657, Japan

**Keywords:** beef cattle, genetic parameter, resilience, selection, uniformity

## Abstract

Economic incentives have increased the demand for uniformity in beef production, making it a valuable phenotype for genetic studies. Genetic heterogeneity of residual variance suggests that variability around the mean may have a genetic component and could potentially respond to selection. However, evidence for genetic control of residual variance in beef cattle remains limited. The objectives of this study were to 1) investigate genetic heterogeneity of residual variances for birth weight (BW), weaning weight (WW), and yearling weight (YW) in American Angus cattle, and 2) compare models for genetic homogeneity (M1) versus genetic heterogeneity of residual variance, including a double hierarchical generalized linear model (DHGLM, M2) and a genetically structured environmental variance model (M3). A total of 75,000 BW, 74,975 WW, and 49,803 YW records were analyzed. Genetic parameters were estimated using average information restricted maximum likelihood for models M1 and M2, whereas model M3 employed Markov chain Monte Carlo within a Bayesian framework. We found evidence of genetic variation in residual variance for all traits, although the heritability estimates for residual variance were low, ranging from 0.004 to 0.01. Estimates of the genetic coefficient of variation for residual variance ranged from 0.38 to 0.62 for BW, 0.09 to 0.25 for WW, and 0.07 to 0.25 for YW, indicating potential for selection to reduce variability. Genetic correlations between mean and residual variance were negative for BW (−0.48 in M2 and −0.49 in M3) and positive for WW and YW. These findings indicate that the implications of selection for uniformity are trait-specific. Body weight should be maintained within an optimal range, whereas selection for increased WW and YW may lead to greater variability unless residual variance is incorporated into selection decisions. Overall, our results indicate the feasibility of reducing variability through selection, representing a first step in integrating growth trait uniformity into breeding goals for beef cattle.

## Teaser

Our study shows that growth uniformity in beef cattle has a genetic basis and can potentially be incorporated into breeding programs, opening the door to more predictable and resilient beef production systems.

## Introduction

Genetic improvement of beef cattle considers several traits of economic importance, such as live weight traits (ie birth, weaning, and yearling weights) as indicators of growth performance (Archer and Bergh 2000; Koknaroglu et al. 2005). Understanding the factors that determine variation in these phenotypes has been of ongoing interest to geneticists and animal breeders because variability is the basis for breeding programs, as genetic variability underpins selection response and long-term progress.

Genetic variation is essential not only for sustained improvement across generations but also for adapting animals to diverse production environments and evolving market demands (Notter 1999). In beef production systems, however, uniformity of performance has become increasingly important alongside average productivity. Market incentives frequently reward producers for delivering homogeneous groups of animals that meet specific carcass and weight specifications (Hennessy 2005; Merks et al. 2012; de Oliveira et al. 2017). Consequently, variability in growth traits can directly translate into economic losses through increased management costs, inefficient grouping strategies, longer feeding periods, and penalties associated with animals falling outside target specifications (Murray 1980; Thonney et al. 1981; Archer and Bergh 2000). Beyond economics, lack of uniformity has also been associated with increased competition, stress, and inefficiencies in different animal production systems (Milligan et al. 2002; Gilmour et al. 2005).

While genetic uniformity is crucial for economic efficiency, there is also increasing interest in animals that are less sensitive to environmental stressors. Livestock are continuously exposed to challenges such as pathogens, heat stress, disease outbreaks, management interventions, and resource limitations. Animals that are minimally affected by these challenges or can rapidly recover are referred to as resilient (Berghof et al. 2019a; Casto-Rebollo et al. 2023). From a breeding perspective, resilience and uniformity are closely related, as both reflect how animals respond to environmental variation. Animals that exhibit more consistent performance across environments are not only more predictable but also require fewer interventions, thereby improving overall system efficiency. As a result, breeding strategies that reduce variability while maintaining or improving mean performance are of increasing interest for commercial operations. One way to support this goal is through models that capture not only average performance but also consistency and sensitivity to environmental variability.

Quantitative genetic models often assume homogeneous residual variance across genotypes, implying that genetic differences affect only the mean performance of traits (Falconer and Mackay 1996). However, a growing body of evidence indicates that residual variance itself may be under genetic control, meaning that some genotypes are inherently more variable (or more stable) than others (Mackay and Lyman 2005; Mulder et al. 2007). Earlier work also demonstrated that heterogeneity in variance across groups such as herds, management units, or treatments can influence genetic evaluation (Hill 1984; Gianola 1986; and Foulley et al. 1990). More recent studies have extended this concept by showing that differences in residual variance can have a genetic basis, making uniformity a potential target for selection. To account for this, several statistical approaches have been proposed to model genetic heterogeneity of residual variance (Sancristobal-Gaudy et al. 1998; Sorensen and Waagepetersen 2003; Hill and Zhang 2004; Ros et al. 2004; Mulder et al. 2007; Mulder et al. 2009; Felleki et al. 2012).

Despite these advances, empirical evidence for genetic control of residual variance in beef cattle growth traits remains limited, particularly in widely used cattle commercial populations (Neves et al. 2011, 2012; Iung et al. 2017). Importantly, there is a lack of studies evaluating genetic heterogeneity of residual variance in American Angus cattle, despite its major role in global beef production. In this sense, the objectives of this study were 1) to investigate the genetic heterogeneity of residual variance in the pedigree level for growth traits in American Angus cattle, and 2) to compare the results of different genetic heterogeneity models.

## Material and methods

### Ethics statement

The datasets used were obtained from pre-existing databases (American Angus Association and Angus Genetics Inc., Saint Joseph, MO, USA). Therefore, Animal Care and Use Committee approval was not required for this study.

### Dataset

The dataset was provided by Angus Genetics Inc. and included phenotypic information for three growth traits: birth weight (BW), weaning weight (WW), and yearling weight (YW). During data cleaning, only records falling within three standard deviations of the overall mean for each trait were retained for analysis. Descriptive statistics of the cleaned data are presented in Table 1.

**Table 1:** Descriptive statistics for growth traits.

Trait	^1^N	Minimum (kg)	Maximum (kg)	Mean±SD	Skewness	Kurtosis
**Birth weight**	75,000	34.00	63.05	53.31 ± 4.23	−0.24	−0.02
**Weaning weight**	74,975	226.80	483.71	291.21 ± 37.12	0.67	0.52
**Yearling weight**	49,803	577.00	725.75	669.38 ± 28.20	0.01	−0.72

1 **N= Number of records**

WW was measured at approximately 208 d of age (120–280 d), and YW at approximately 392 d (315–618 d). WW was adjusted for age according to the Beef Improvement Guidelines (Beef Improvement Federation 2016). Animals were born between 1986 and 2013 and raised in different ecoregions of the United States. Contemporary groups (CG) were defined as the concatenation of sex, herd, year, and season of birth, as provided by Angus Genetics Inc. as part of their standard genetic evaluation pipeline. CG with at least 100 animals for YW and 200 animals for BW and WW were retained for analysis. Table 2 shows the data structure after data cleaning.

**Table 2:** Data structure for growth traits.

	Trait		
	BW	WW	YW
**Number of records**	75,000	74,975	49,803
**Number of animals in pedigree**	133,405	125,714	90,122
**Number of contemporary groups**	318	307	422
**Number of sires**	3,967	3,315	3,652
**Number of dams**	54,438	47,424	36,667
**Average number of progenies per sire**	18.91	22.62	13.64
**Average number of animals per CG**	235.85	244.22	118.02

**BW, birth weight; WW, weaning weight; YW, yearling weight; CG, contemporary group.**

### Estimation of variance components

Three models were fitted to estimate genetic parameters using restricted maximum likelihood (REML) and Bayesian approaches. The first was a homoscedastic best linear unbiased prediction model (M1), which is the traditional additive genetic model including the assumption of homogeneity of residual variance, where REML was used to infer variance components. The second (M2) and third (M3) models assumed that residual variance is heterogeneous and partially under genetic control. A REML-based double hierarchical generalized linear model (DHGLM) (Rönnegård et al. 2010) and a Bayesian genetically structured model (SanCristobal-Gaudy et al. 1998) were used in M2 and M3, respectively. Model 1 and M2 were implemented using the average information REML module of the DMU package (Madsen et al. 2006), while M3 was implemented using the GSEVM software (Ibáñez-Escriche et al. 2010).

### Homoscedastic residual variance model (M1)

The following standard animal model for BW and WW, assuming homogeneous residual variance, was fitted for M1:


$$y=\mathrm{Xb}+Z_{1}a+Z_{2}m+\mathrm{Wc}+e$$

where y is a vector of observations (BW or WW); b is a vector of fixed effects including CGs (as previously described) and dam age at calving as a linear covariate; a is a vector of direct additive genetic effects; m is a vector of maternal additive genetic effects; c is a vector of maternal permanent environmental effects; e is a vector of residuals; and$\;X,\;Z_{1},\;Z_{2}$, and W are incidence matrices relating b, a, m, and c to  y, respectively. Here, the age of dams ranged from 2 to 11 years. It was assumed that:


$$E=[\begin{array}{c} y\\ a\\ m\\ c\\ e \end{array}]=[\begin{array}{c} Xb\\ 0\\ 0\\ 0\\ 0 \end{array}];\;V[\begin{array}{c} a\\ m\\ c\\ e \end{array}]=[\begin{array}{ccc} G⨂A & 0 & 0\\ 0 & {I_{d}\sigma }_{c}^{2} & 0\\ 0 & 0 & {I_{n}\sigma }_{e}^{2} \end{array}]$$

where$\;G=[\begin{array}{cc} \sigma_{a}^{2} & \sigma_{\mathrm{am}}\\ \sigma_{\mathrm{am}} & \sigma_{m}^{2} \end{array}]\;$is a matrix of genetic covariances between direct and maternal effects;$\;\sigma_{a}^{2}\;$is the additive genetic variance;$\;\sigma_{m}^{2}\;$is the maternal additive genetic variance;$\;\sigma_{\mathrm{am}}\;$is the covariance between maternal and direct additive genetic effects; A  is the pedigree-based relationship matrix;$\;I_{d}\;$is the identity matrix of order equal to the number of dams;$\;\sigma_{c}^{2}\;$is the maternal permanent environmental variance;$\;I_{n}\;$is the identity matrix equal to the number of observations; and$\;\sigma_{e}^{2}\;$ is the residual variance.

The following standard animal model was used for YW:


y=Xb+Za+e

where y is a vector of phenotypes, X  is an incidence matrix of fixed effects; b is a vector of fixed effects (CGs including animals born on the same farm, year, and management group at yearling, and age at yearling as a linear covariate); Z is an incidence matrix relating individuals to phenotypic records; a is the additive genetic variance with$\;a\;∼N(0,A\sigma_{a}^{2})$; and e is a vector of residuals, assuming$\;e\;∼N(0,I\sigma_{e}^{2})$.

### Heterogeneous residual variance models

#### Model (M2)

A DHGLM was used in M2, which jointly models both the mean and the residual variance of the trait. This approach is an extension of generalized linear models (Lee and Nelder 1996) by jointly modeling the mean and residual variance of the trait. It enables estimation of genetic and environmental contributions to both the trait mean and its within-individual variance. Residual variance is modeled as a secondary trait, incorporating its own explanatory variables and random effects. More specifically, DHGLM assumes a gamma distribution for the squared residuals, which is suitable for modeling positively skewed data (Rönnegård et al. 2010). During each iteration, the model fits a gamma distribution to the squared residuals and updates the parameter estimates. This approach addresses non-normality by modeling squared residuals rather than the observed values.

Later, the DHGLM approach was extended by simultaneously fitting the trait (also referred to as the mean) and residual variance within a bivariate model as follows (Felleki et al. 2012):


$$\left[\begin{array}{c} y\\ \psi \end{array}]=[\begin{array}{cc} X & 0\\ 0 & X_{V} \end{array}][\begin{array}{c} b\\ b_{v} \end{array}]+[\begin{array}{cc} Z & 0\\ 0 & Z_{V} \end{array}][\begin{array}{c} a\\ a_{v} \end{array}]+[\begin{array}{cc} W & 0\\ 0 & W_{V} \end{array}][\begin{array}{c} c\\ c_{v} \end{array}]+[\begin{array}{c} e\\ e_{v} \end{array}\right]$$

where  y is the vector of observations (BW, WW, or YW), ψ  is the linear predictor of the residual variance on the log scale;$\;X,\;X_{V}$ are incidence matrices for fixed effects b and $b_{v}$ affecting the mean and residual variance, respectively; Z and $Z_{V}$ link additive genetic effects a  and$\;a_{v}\;$to the mean and variance parts; W and$\;W_{V}\;$are incidence matrices for permanent environmental effects c and$\;c_{v}\;$in both mean and residual variance; and e and$\;e_{v}\;$are residuals for the mean and residual variance parts, respectively.

The linear predictor for residual variance,$\;{\psi \;}_{i}$, is defined as:


$${\psi \;}_{i}=\log\left({\hat{\sigma }}_{e_{i}}^{2})\;+\;(\{[\;{\hat{e}}_{i}^{2}/(1-h_{i})]-\;{\hat{\sigma }}_{e_{i}}^{2}\}/{\hat{\sigma }}_{e_{i}}^{2}\right)$$

where$\;{\hat{\sigma }}_{e_{i}}^{2}\;$is the predicted residual variance for observation i from the previous iteration;$\;{\hat{e}}_{i}^{2}\;$is the squared residual from the mean model estimated for observation i; and$\;h_{i}\;$is the leverage for observation i that is the$\;i^{\mathrm{th}}\;$diagonal element of the hat matrix (Hoaglin and Welsch 1978). The fixed effects in the model are the same as those described previously for M1. Further, it was assumed that:


$$\left[\begin{array}{c} a\\ a_{v} \end{array}]∼\;N(0,[\begin{array}{cc} \sigma_{a}^{2} & \sigma_{a,a_{v}}\\ \sigma_{a,a_{v}} & \sigma_{a_{v}}^{2} \end{array}]⨂\;A\right)$$

where ⨂ denotes the Kronecker product and A  is the numerator relationship matrix. Also,


$$\left[\begin{array}{c} e\\ e_{v} \end{array}]∼\;N(0,[\begin{array}{cc} \Omega^{-1}\sigma_{e}^{2} & 0\\ 0 & \Omega_{v}^{-1}\sigma_{e_{v}}^{2} \end{array}]⨂\;I\right)$$

where$\;\Omega \;\mathrm{and}\;\Omega_{v}\;$are diagonal matrices defined as $\Omega =\mathrm{diag}({\hat{\psi }}^{-1})$ and$\;\Omega_{v}=\mathrm{diag}(\frac{1-h}{2})$, respectively, and are the residual variance per observation, because$\;\sigma_{e}^{2}\;$and$\;\sigma_{e_{v}}^{2}\;$are scaling variances that are expected to be equal to 1;$\;\hat{\psi }\;$is the updated estimate of the residual variance for each observation at a given iteration, and h refers to the vector of leverage values for all individuals in the dataset. The parameters ψ, Ω, and$\;\Omega_{v}\;$were updated in each iteration until the algorithm converged. Convergence was assumed when the relative difference in the estimates of the variance components between iterations was less than$\;10^{-6}$.

#### Model 3 (M3)

Model 3 is a genetically structured variance model proposed by SanCristobal-Gaudy et al. (1998), which assumes that residual variance is heterogeneous and partially under genetic control. The residual variance is modeled as an exponential function of covariates and random effects. Model 3 for BW and WW were:


$$y_{i}=x_{i}b+z_{i}a+w_{i}c+e^{\frac{1}{2}(x_{i}b^{*}+z_{i}a^{*}+w_{i}c^{*})}\varepsilon_{i}$$

And for YW:


$$y_{i}=x_{i}b+z_{i}a+e^{\frac{1}{2}(x_{i}b^{*}+z_{i}a^{*})}\varepsilon_{i}$$

where$\;x_{i}$,$\;z_{i}\;$and$\;w_{i}\;$are vectors of covariates for fixed, genetic, and permanent environmental effects; b and$\;b^{*}\;$are the vectors of fixed effects for the mean and residual variance, respectively; a and$\;a^{*}\;$are additive genetic effects for the mean and residual variance; c and$\;c^{*}\;$represent the vectors associated with permanent environmental effects (BW and WW only), and$\;\varepsilon_{i}\;$is the is standard normal error.

The exponential function models residual variance as a function of both genetic and environmental predictors. The additive genetic effects a and$\;a^{*}\;$are modeled as jointly normally distributed random variables:


$$\left[\begin{array}{c} a\\ a^{*} \end{array}]∼\;N([\begin{array}{c} 0\\ 0 \end{array}],[\begin{array}{cc} \sigma_{a}^{2} & \rho \sigma_{a}\sigma_{a^{*}}\\ \rho \sigma_{a}\sigma_{a^{*}} & \sigma_{a^{*}}^{2} \end{array}]⨂\;A\right)$$

where$\;\sigma_{a}^{2}\;$is the additive genetic variance;$\;\sigma_{a^{*}}^{2}\;$is the additive genetic variance that influences the residual variance of the trait; ρ is the correlation coefficient between$\;\sigma_{a}^{2}\;$and$\;\sigma_{a^{*}}^{2}$. Also,$\;\sigma_{a^{*}}^{2}\;$was directly compared to$\;\sigma_{a_{v}}^{2}\;$in M2 in the following sections.

Lastly, c and$\;c^{*}\;$were assumed to be independent:


$$c∼N(0,I_{c}\sigma_{c}^{2})$$

$$c^{*}∼N(0,I_{c}\sigma_{c^{*}}^{2})$$

where$\;I_{c}\;$is an identity matrix of the same order as the number of animals recorded for BW and WW, and$\;\sigma_{c}^{2}\;$and$\;\sigma_{c^{*}}^{2}\;$are the permanent environmental variances affecting the mean and residual variance, respectively. Model 3 was implemented using the GSEVM program (Ibáñez-Escriche et al. 2010). For each trait, the Markov chain Monte Carlo run consisted of 500,000 to 1,000,000 iterations with a burn-in period of 50,000 to 100,00 iterations. The Geweke criterion (Geweke 1991) was used to diagnose the chains at a 5% significance level using the boa package (Smith 2007) in R (R Core Team 2021).

### Genetic parameters for the mean and residual variance

Variance components from M1, M2, and M3 were used to estimate genetic parameters for BW, WW, and YW. For M2, two genetic parameters were calculated for the residual variance: the heritability of the residual variance ($h_{v}^{2}$) and the genetic coefficient of variation,$\;GCV_{E}\;$(Mulder et al. 2007). The heritability of residual variance$\;h_{v}^{2}\;$ reflects the accuracy of estimated breeding values for variability and was calculated as follows*:*


$$h_{v}^{2}=\frac{\sigma_{a_{v,\mathrm{add}}}^{2}}{\left(2\sigma_{p}^{4}+3\sigma_{a_{v,\mathrm{add}}}^{2}\right)}$$

where$\;\sigma_{a_{v,\mathrm{add}}}^{2}\;$is the additive genetic variance estimated for the residual variance on the additive scale and$\;\sigma_{p}^{4}\;$is the squared phenotypic variance. Here,$\;\sigma_{a_{v,\mathrm{add}}}^{2}$and the standard error of $h_{v}^{2}\;$were obtained according to Mulder et al. (2016).$\;GCV_{E}\;$evaluates the potential response to selection for residual variance as follows:


$$GCV_{E}=\frac{\sigma_{a_{v,\mathrm{add}}}}{\bar{{\bar{\sigma }}_{\hat{e}}^{2}}}$$

where$\;\sigma_{a_{v,\mathrm{add}}}\;$is the standard deviation of the additive genetic variance for the residual variance on the additive scale. The$\;\mathrm{GC}V_{E}\;$was calculated as described in Mulder et al. (2007) and Hill et al. (2010).

Under M3**,** heritability estimates of the mean of BW, WW, and YW are not unique because they depend on the value of the residual variance, which is influenced by different levels of environmental effects (represented by$\;b^{*}$) (Ibáñez-Escriche et al. 2008; Formoso-Rafferty et al. 2017):


Var[yi|b, b*]=σa2+σc2+exp⁡((Xb*)i+σa*22+σc*22).

Therefore, heritability estimates ($h_{i}^{2}$) were obtained for each combination of levels of fixed effects$\;(Xb^{*}{)}_{i}\;$(Sorensen and Waagepetersen 2003; Ros et al. 2004)


$$h_{i}^{2}=\frac{\sigma_{a}^{2}}{\sigma_{a}^{2}+\sigma_{c}^{2}+\exp((Xb^{*}{)}_{i}+\frac{\sigma_{a*}^{2}}{2}+\frac{\sigma_{c*}^{2}}{2})}.$$

Lastly, we obtained the genetic correlation between the mean and the residual variance ($r_{\mathrm{mv}}$). This is a genetic parameter that guides selection for uniformity, and it ranges from −1.0 to 1.0.

## Results

### Estimates of variance components and genetic parameters

Estimates of variance components, and genetic parameters under the three models for BW, WW, and YW are presented in Table 3.

**Table 3:** Variance component estimates for mean and residual variances of growth traits.

Trait	Model	$\sigma_{a}^{2}$	$\sigma_{m}^{2}$	$\sigma_{c}^{2}$	$\sigma_{e}^{2}$	^2^ $\sigma_{a*}^{2}$	$\sigma_{c*}^{2}$	$h^{2}$	$h_{v}^{2}$	${\mathrm{GCV}}_{E}$	$r_{\mathrm{mv}}$
**BW**	M1	27.17 (0.90)	4.03 (0.72)	$0.17\times 10^{-5}$ (0.71)	30.16 (0.61)	–	–	0.44 (0.01)	–	–	–
	M2	15.50 (3.64)	–	1.11 (0.62)	22.15 (1.84)	0.13 (0.01)	1.39 (0.24)	0.39 (0.07)	$4.00\times 10^{-3}$ $\left(2.00\times 10^{-4}\right)$	0.62 (0.06)	−0.48 (0.03)
	^1^M3	18.77 (3.48)	–	22.35 (1.90)	–	0.14 (0.01)	0.57 (0.31)	0.39 (0.04)	–	0.38 (0.05)	−0.49 (0.02)
**WW**	M1	791.78 (35.50)	154.89 (28.97)	146.08 (28.87)	1,876.31 (25.65)	–	–	0.26 (0.01)	–	–	–
	M2	773.05 (136.37)	–	422.20 (47.07)	2,329.70 (69.68)	0.07 (0.01)	10.83 (5.45)	0.21 (0.03)	0.01 ($1.00\times 10^{-3}$)	0.09 (0.03)	0.54 (0.06)
	^1^M3	780.96 (139.61)	–	3,648.35 (246.83)	–	0.06 ($2.00\times 10^{-3}$)	14.07 (6.87)	0.23 (0.02)	–	0.25 (0.04)	0.61 (0.07)
**YW**	M1	1,685.43 (11.17)	–	–	4,333.96 (60.37)	–	–	0.27 (0.08)	–	–	–
	M2	1,641.32 (15.01)	–	–	4,577.84 (85.76)	0.08 ($8.92\times 10^{-3}$)	–	0.26 (0.05)	0.01 ($3.00\times 10^{-3}$)	0.07 (0.02)	0.72 (0.09)
	^1^M3	1,633.66 (17.03)	–	–	–	0.06 (0.01)	–	0.26 (0.06)	–	0.25 (0.03)	0.39 (0.04)

**BW, birth weight; WW, weaning weight; YW, yearling weight; M1, Homoscedastic residual variance Model; M2, Heteroscedastic model (DHGLM); M3, Heteroscedastic model (structured model).**  $\sigma_{a}^{2}\;\mathrm{and}\;\sigma_{\mathrm{av}}^{2}\;\mathrm{are}\;\mathrm{the}\;\mathrm{additive}\;\mathrm{genetic}\;\mathrm{variance}\;\mathrm{for}\;\mathrm{the}\;\mathrm{means}\;\mathrm{and}\;\mathrm{residual}\;\mathrm{variances}.\sigma_{c}^{2}\;\mathrm{and}\;\sigma_{c*}^{2}\;a\mathrm{re}\;\mathrm{the}\mathrm{permanent}\;\mathrm{environmental}\;\mathrm{variances}\;\mathrm{for}\;\mathrm{the}\;\mathrm{means}\;\mathrm{and}\;\mathrm{residual}\;\mathrm{variances}.\;\sigma_{m}^{2}\;\mathrm{is}\;\mathrm{the}\;\mathrm{maternal}\;\mathrm{genetic}\;\mathrm{variance}.\sigma_{e}^{2}\;\mathrm{is}\;\mathrm{the}\;\mathrm{residual}\;\mathrm{variance}.h^{2}\;\mathrm{and}\;h_{v}^{2}\;\mathrm{are}\;\mathrm{the}\;\mathrm{heritability}\;\mathrm{estimates}\;\mathrm{for}\;\mathrm{the}\;\mathrm{means}\;\mathrm{and}\;\mathrm{residual}\;\mathrm{variances}.\;{\mathrm{GCV}}_{E}\;\mathrm{is}\;\mathrm{the}\;\mathrm{genetic}\;\mathrm{coefficient}\;\mathrm{of}\;\mathrm{variation}\;\mathrm{for}\;\mathrm{the}\;\mathrm{residual}\mathrm{variance};\;r_{\mathrm{mv}}$  **is the genetic correlation between the estimated breeding values of the means and residual variances; standard errors are given in parentheses.**

1 **Mean and SE (in parentheses) of the marginal posterior distribution.**

2 **For M3,**  $\sigma_{\mathrm{av}}^{2}\;\mathrm{represents}\;\sigma_{a^{*}}^{2}$

### Homoscedastic residual variance model (M1)

**Figure 1:**
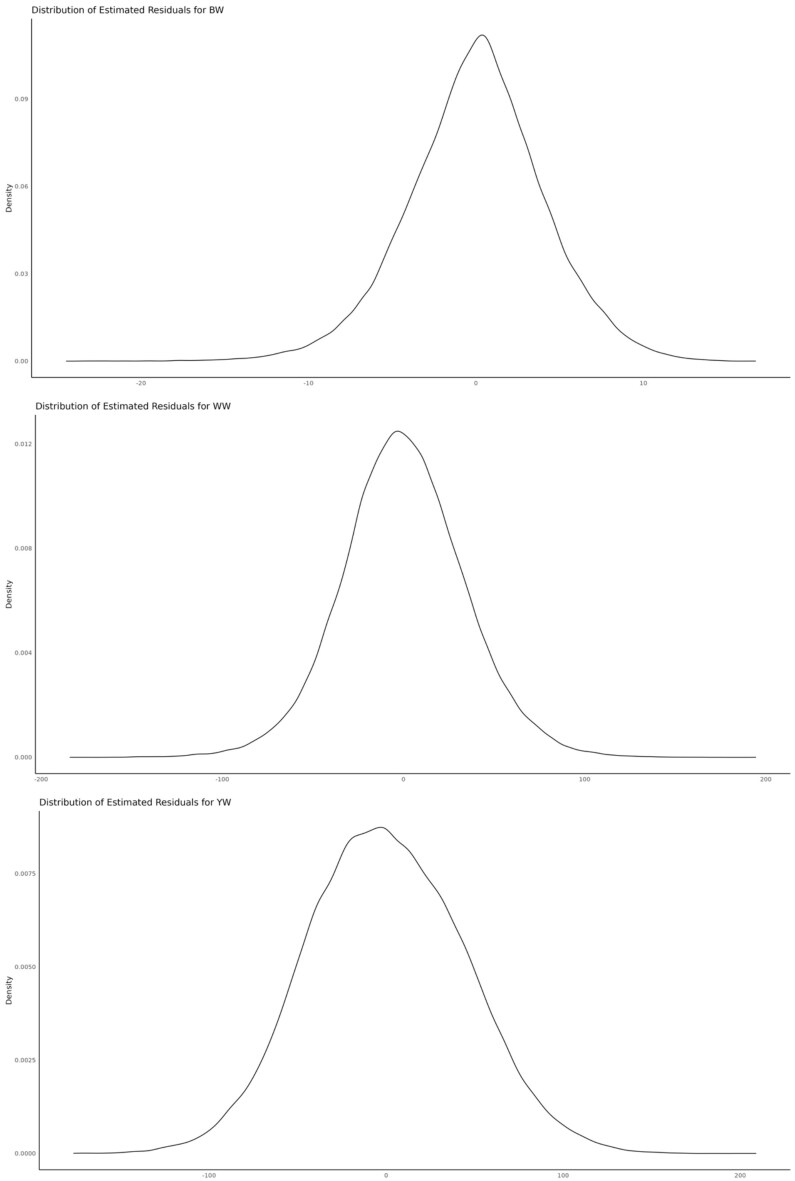
Distribution of estimated residuals based on the homoscedastic model (M1) for body weight (BW), weaning weight (WW), and yearling weight (YW).

BW exhibited the highest heritability (0.44), while estimates for WW and YW were 0.21 and 0.26, respectively. These results serve as a baseline for evaluating changes under models that account for residual variance heterogeneity. The estimated distributions of the residuals are shown in Figure 1.

### Heterogeneous residual variance models (M2 and M3)

Regarding the additive genetic variance for the mean ($\sigma_{a}^{2})$, M2 and M3 gave smaller estimates of$\;\sigma_{a}^{2}\;$than M1 for BW (27.17 for M1, 15.50 for M2, and 18.77 for M3), WW (791.78 for M1, 773.80 for M2, and 780.96), and YW (1,685.43 for M1, 1,641.32 for M2, and 1,633.66 for M3). Compared to M1, both M2 and M3 showed reduced estimates of additive genetic variance for the mean ($\sigma_{a}^{2}$) across all traits, indicating the influence of modeling heterogeneous residual variance.

The parameters influencing the genetic control of the residual variances were estimated with M2 and M3. Estimates of the genetic variance for residual variance ($\sigma_{\mathrm{av}}^{2}\;$in M2 and$\;\sigma_{a*}^{2}\;$in M3) were generally low to moderate. In M2,$\;\sigma_{\mathrm{av}}^{2}\;$was 0.13 for BW, 0.07 for WW, and 0.08 for YW. For M3, the posterior means of$\;\sigma_{a*}^{2}\;$were 0.14 for BW, 0.06 for WW, and 0.06 for YW. BW showed slightly higher genetic variance in residual variance than WW and YW.

Heritability estimates of residual variance ($h_{v}^{2}$) were low (<0.1), consistent with prior literature, but sufficient to support potential selection. Estimates of$\;h_{v}^{2}\;$were 0.004 for BW, 0.01 for WW, and 0.01 for YW, respectively. Overall, estimates of $\mathrm{GC}V_{E}\;$were low to moderate for all traits in M2. For example,$\;\mathrm{GC}V_{E}\;$estimates were 0.62 for BW, 0.09 for WW, and 0.07 for YW. Estimates of$\;\mathrm{GC}V_{E}\;$from M3 were 0.38 for BW, 0.25 for WW, and 0.25 for YW. With the exception of BW, M3 presented the higher$\;\mathrm{GC}V_{E}\;$estimates than M2.$\;\mathrm{GC}V_{E}\;$values suggested that despite low$\;h_{v}^{2}$, there is genetic potential for reducing variability, particularly in BW.

Lastly, the genetic correlation between the additive values of the mean and variance ($r_{\mathrm{mv}}$) was calculated for M2 and M3. Overall, positive estimates were observed, except for BW in M2 (−0.48) and M3 (−0.49). The negative genetic correlation in BW suggests potential to increase mean performance while reducing residual variance. The$\;r_{\mathrm{mv}}\;$estimates in M2 for WW and YW were 0.54, and 0.72, respectively. For M3, the$\;r_{\mathrm{mv}}\;$estimates were 0.61 for WW, and 0.39 for YW, respectively. The positive genetic correlations found for WW and YW indicate that selection for increased mean might lead to increased residual variance unless accounted for explicitly.

## Discussion

### Genetic variances and parameters for the mean

To our knowledge, this is one of the first studies of genetic heterogeneity of residual variance in American Angus cattle, and to compare DHGLM and structured approaches to estimate genetic variance for growth weight uniformity, constituting the first steps to investigate the possibility of including these traits in breeding goals. The$\;h^{2}\;$estimates in the three models for BW (0.44 in M1, 0.39 in M2, and 0.39 in M3) obtained in this study were lower than those observed by Smith et al. (2022) (0.51) and slightly higher in M1 than those of Lourenco et al. (2015) (0.41), and similar to the estimates in M2 and M3. The$\;h^{2}\;$estimates for WW (0.26 in M1, 0.21 in M2, and 0.23 in M3) were similar to those reported by Smith et al. (2022) (0.25) and Lourenco et al. (2015) (0.20). For YW, our estimates (0.27 in M1, 0.26 in M2, and 0.26 in M3) were lower than the genomic heritability estimates reported by Smith et al. (2022) (0.42) in Red Angus cattle and Schiermiester et al. (2015) (0.38–0.39) in a multibreed dataset including Angus cattle. The lower$\;h^{2}\;$values obtained for YW could be explained by two reasons: 1) only a subset of the whole American Angus population and 2) pedigree information was used in current the analysis. Therefore, there is a possibility that the dataset for YW used in this study may not be a representative sample of the national herd.

### Genetic variances and parameters for the variance

Models 2 and M3 assume that there are genes controlling not only the mean of a trait but also its residual variance (SanCristobal-Gaudy et al. 1998). Our results show the presence of additive genetic variances for the means ($\sigma_{a}^{2}$) of BW, WW, and YW and their variances ($\sigma_{\mathrm{av}}^{2}\;$in M2 and$\;\sigma_{a*}^{2}\;$in M3) in both models.

Low estimates of$\;h_{v}^{2}\;$were observed for all traits, consistent with the range reported in the literature. For example, Neves et al. (2011) performed genetic heterogeneity of residual variance analysis for over fifteen traits in Nellore cattle by analyzing the log squared residuals of each phenotypic observation using an animal model. Estimates of genetic variances and parameters were obtained using a two-step approach consisting of first fitting a model to the mean and then using the log-transformed squared estimated residuals as a measure of residual variance in the second step. For BW, they reported pedigree-based$\;h_{v}^{2}\;$estimates of 0.09 (±0.01). Using DHGLM in pigs, Sell-Kubiak et al. (2015a) found$\;h_{v}^{2}\;$estimates for BW ranging from 0.008 to 0.01. In chickens, Yousefi Zonuz et al. (2019) reported$\;h_{v}^{2}\;$for BW ranging from 0.08 (±0.002) to 0.09 (±0.003), and Sae-Lim et al. (2018) showed that$\;h_{v}^{2}\;$for BW in sheep was 0.07.

To our knowledge, this is the first study to report the extent of genetic control of residual variance for WW in livestock species. Estimates of$\;h_{v}^{2}\;$obtained were 0.01 (±0.001). The closest trait reported in the literature for cattle is weight gain from birth to weaning. For example, Neves et al. (2011) reported$\;h_{v}^{2}\;$estimates of 0.02 (±0.003) for birth to WW gain. Similarly, Neves et al. (2012) used three different datasets (male, female, and combined) to investigate genetic heterogeneity in the residual variance of various weight traits in Nellore cattle using a two-step approach. The$\;h_{v}^{2}\;$estimates for weight gain from birth to weaning were 0.03 for males, 0.05 for females, and 0.03 for combined data.

YW is a key breeding goal for beef cattle because it reflects the performance of the animal at an age when there is a high volume of animal trade. Our estimates of$\;h_{v}^{2}\;$were 0.01. Neves et al. (2012) reported$\;h_{v}^{2}\;$estimates for YW of 0.03 for females, 0.05 for males, and 0.02 for combined data. More recently, Iung et al. (2017) investigated genetic heterogeneity of residual variance in YW in a Nellore cattle population using a two-step approach and DHGLM for untransformed and Box-Cox-transformed phenotypes. For untransformed data, they reported$\;h_{v}^{2}\;$estimates of 0.01 (±0.002) for females, 0.003 (±0.001) for males, and 0.07 (±0.001) for combined data using the DHGLM approach. After transformation, the$\;h_{v}^{2}\;$estimate was reduced to 0.003 (±0.001) for combined data. The estimates in our study were the same in M2 when comparing the results of Iung et al. (2017) with the data for females and were higher for YW compared to the full data.

The low estimates of$\;h_{v}^{2}\;$obtained in this study and other work in different species (Hill and Mulder 2010; Iung et al. 2020) suggest the need for larger data to predict estimated breeding values for residual variances (Mulder et al. 2007). The primary issue is that$\;h_{v}^{2}\;$in M2 and M3 are defined at the level of individual single records. Estimating variance from single phenotypes can be challenging because the sampling variance of a variance estimate typically exceeds that of a mean, and these sampling problems tend to occur when environmental effects affect variability (Iung et al. 2017).

In addition to statistical challenges, the structure and editing of the data may also influence these estimates. The data editing criteria used in our study should also be considered when interpreting results. Records outside of three standard deviations from the mean were removed, and only CG with a minimum number of records were retained. These criteria may have reduced the representation of extreme phenotypes and smaller management groups. Therefore, estimates of genetic variance for residual variance and related parameters should be interpreted in the context of the edited dataset.

Despite these limitations, previous studies suggest that selective breeding can improve uniformity. For example, early selection experiments have successfully reduced residual variance in body weight and litter size in rabbits (Garreau et al. 2008; Blasco et al. 2017) and BW in mice (Formoso-Rafferty et al. 2016).

### Opportunities for genetic improvement

The$\;\mathrm{GC}V_{E}\;$evaluates the potential response to selection for residual variance, and although$\;h_{v}^{2}\;$estimates were not high in our study,$\;\mathrm{GC}V_{E}\;$estimates show that improving uniformity for BW, WW, and YW through selection is feasible. Estimates of$\;\mathrm{GC}V_{E}\;$in M3 were higher than the estimates in M2 for WW and YW.

For BW, the estimated$\;\mathrm{GC}V_{E}\;$found in our study was higher than that of Neves et al. (2011), who reported$\;\mathrm{GC}V_{E}\;$of 0.69 in Nellore cattle, Sell-Kubiak et al. (2015a) in pigs (0.20–0.24), and Sae-Lim et al. (2018) in sheep (0.58). Our estimates of$\;\mathrm{GC}V_{E}\;$for WW was 0.09 in M2, and 0.25 in M3. Our results are in line with the estimates for weight gain from birth to weaning reported in Neves et al. (2011, 2012). Lastly, our estimates of$\;\mathrm{GC}V_{E}\;$for YW in M2 (0.07) and M3 (0.25) were smaller than those in Neves et al. (2012), which were 0.43 for females, 0.79 for males, and 0.43 for the combined data. The estimates in Iung et al. (2017) were smaller than most of our results and those of Neves et al. (2012). They reported 0.20 for females, 0.10 for males, and 0.17 for the combined data and 0.12 after Box-Cox transformation.

Our results for$\;\mathrm{GC}V_{E}\;$based on M2 suggest that a change of 1 genetic standard deviation would change the residual variance by 62% for BW, 9% for WW, and 7% for YW. For M3, a change of 1 genetic standard deviation would change the residual variance by 38% for BW, 25% for WW, 25% for YW. The estimates in this study for M2 and M3 were within the range previously reported for other species (Hill and Mulder 2010; Iung et al. 2020). This suggests that the genetic heterogeneity of residual variance is sizeable, indicating the potential for selection to alter residual variance.

The genetic correlation between the mean and residual variance ($r_{\mathrm{mv}})\;$further informs these selection strategies. This parameter ranges from −1.0 to 1.0, and in general, negative values are preferred when the objective is to increase the mean while decreasing variability. When this relationship is unfavorable, both the mean and residual variance should be included in a selection index to achieve the desired response (Mulder et al. 2008; Berghof et al. 2019a). In our study, BW based on M2 and M3 showed a very similar negative moderate genetic correlation (M2: −0.48; M3: −0.49) between the mean and residual variance. The negative$\;r_{\mathrm{mv}}\;$for BW indicates that selection for higher average BW may be associated with reduced variability in BW. In practical terms, this could help produce more uniform calves with more predictable BW. However, BW is a key determinant of calving ease, with heavier calves generally increasing the risk of calving difficulty (ie dystocia). Therefore, this relationship must be interpreted with caution. Even if variability decreases, selection that shifts the population mean for BW upward could still increase dystocia risk if BW exceed an optimal range. In practice, this implies that BW should be targeted for maintenance within an optimal range rather than directional increase. Estimates of$\;r_{\mathrm{mv}}\;$for BW reported in the literature are 0.42 in cattle (Neves et al. 2011), 0.39 in sheep (Sae-Lim et al. 2018), 0.53 to 0.62 in chickens (Sell-Kubiak et al. 2015a, 2015b), −0.02 for weight gain from birth to weaning in beef cattle (Neves et al. 2011), and 0.21 to 0.27 for the same trait in beef cattle (Neves et al. 2012).

In contrast with$\;r_{\mathrm{mv}}\;$estimates for BW, all$\;r_{\mathrm{mv}}\;$for WW and YW were positive. For WW and YW, breeding programs generally aim to increase mean performance to improve growth and productivity. In this context, selection for increased mean performance is expected to be accompanied by increased residual variability, which represents a less favorable scenario for breeding programs, as it creates an inherent trade-off between improving average performance and maintaining uniformity. In practical terms, selection based solely on estimated breeding values for WW or YW may result in animals with superior growth potential but increased variability in performance. Therefore, selection strategies based solely on estimated breeding values for the mean may lead to less predictable outcomes. To address this, selection indices offer a practical approach to managing this trade-off. By assigning appropriate economic weights to both the mean and residual variance, it is possible to balance gains in growth with acceptable levels of variability. However, the magnitude of the positive correlation determines how restrictive this trade-off becomes, as stronger correlations may limit the simultaneous improvement of both traits and require more conservative selection decisions. Therefore, although selection index can help mitigate these unfavorable relationships, it may come at the cost of reduced genetic gain in the mean.

The findings of this study offer important insights for the genetic evaluation of beef cattle. The moderate negative genetic correlations observed between mean and residual variance in BW suggest that animals with superior average performance may also exhibit reduced phenotypic variability, an advantageous combination for commercial systems seeking predictability. Conversely, for traits where the correlations were positive, selection on the mean alone may inadvertently increase variability, potentially leading to inconsistent performances. Incorporating genetic heterogeneity of residual variance into breeding programs could improve the identification of animals that not only perform well on average but also do so more uniformly across different environments. This is particularly relevant in large-scale operations where animals experience a wide range of management conditions. Ultimately, selection strategies that integrate both mean performance and variability metrics can enhance robustness, reduce management burden, and align better with industry demands for consistency and efficiency.

### REML and Bayesian approaches

Modeling genetic heterogeneity of residual variance assumes the presence of genes that influence mean performance and variability under changing environmental conditions. SanCristobal-Gaudy et al. (1998) proposed a model based on the expectation maximization algorithm to estimate genetic parameters. Since then, alternative models have been introduced, including the two-step approach (Garreau et al. 2008; Mulder et al. 2009), DHGLM (Rönnegård et al. 2010; Felleki et al. 2012), and the structured approach (Sorensen and Waagepetersen 2003).

In our study, we assessed variance components and genetic parameters for residual variance using REML (DHGML) and Bayesian (structured) approaches. The estimates of$\;\sigma_{\mathrm{av}\;}^{2}$in the REML approach was slightly higher than the Bayesian approach ($\sigma_{a*}^{2}$) for WW (M2: 0.07; M3: 0.06) and YW (M2: 0.08; M3: 0.06). In contrast, for BW, the Bayesian approach showed a slightly higher estimate of$\;\sigma_{a*\;}^{2}\;$compared to the REML approach$\;(\sigma_{\mathrm{av}}^{2})$(M2: 0.13; M3: 0.14).

Due to software limitations, the software used for the Bayesian approach was not able to account for maternal effects for BW and WW, as it can only fit additive and permanent environmental effects. In this sense, to make fair comparisons between approaches, maternal effects were not included in M2. However, we assumed that the permanent environmental effects (c and$\;c^{*}$) captured a large portion of the maternal influence, as suggested by Ibáñez-Escriche et al. (2008). In their study, on environmental variability of weight gain in mice, the authors found that litter and maternal effects were largely confounded and could not be clearly separated, indicating that the maternal component was not entirely excluded but instead absorbed by the permanent environmental term. In our study, although permanent environmental effects were modeled, it is possible that some portion of the genetic variance for residual variance reflects unmodeled maternal contributions. Future work should explore models that include both maternal and residual variance effects to better disentangle their contributions and improve the interpretation of genetic control over residual variance.

Estimates of$\;\mathrm{GC}V_{E}\;$and$\;r_{\mathrm{mv}}\;$varied between the two approaches but were within the ranges reported in the literature for different species. Studies using both REML and Bayesian approaches on the same data have reported similar estimates of$\;\sigma_{\mathrm{av}}^{2}\;$and$\;\sigma_{a*}^{2}\;$(Wolc et al. 2009; Rönnegård et al. 2010). However, Gutiérrez et al. (2006) found different estimates between REML and Bayesian approaches in the same data when examining genetic heterogeneity for litter size, litter weight, and individual BW in mice. Further studies with real and simulated data should evaluate these methods in repeated observations on the same individual or with larger families (Iung et al. 2020).

In terms of computational time in this study, M2 took approximately 8 h to complete for each trait, while M3 took an average of 16 h per trait when run on a system equipped with an Intel(R) Xeon(R) Gold 6248R CPU @ 3.00 GHz and 1.5 TB of memory. The Bayesian approach is computationally expensive due to its complex sampling techniques, using combinations of Gibbs sampling and Metropolis-Hastings algorithms, which are essential for estimating all parameters because the full conditional distributions do not conform to standard forms (Sorensen and Waagepetersen 2003; Ibáñez-Escriche et al. 2010).

### Implications for breeding

Genetic heterogeneity of residual variance refers to the idea that, in addition to the genetic regulation of a trait’s mean, the variability around that mean (residual variance) may also be under genetic control. This opens the door for selection strategies that target not only higher average performance but also greater predictability and consistency across production environments or repeated measures. However, the direction of selection depends on the breeding objective of each trait. For example, BW is typically managed within an optimal range to avoid dystocia, whereas growth traits such as WW and YW are generally selected to increase performance within economically desirable limits. In this framework, incorporating residual variance into selection decisions may help improve both performance and uniformity across animals. Although the estimates of$\;h_{v}^{2}\;$were low, they indicate that genetic improvement in residual variance is feasible. Traits with low heritability can respond to selection, particularly when large datasets are available, and selection intensity is sufficiently high. In this context,$\;\mathrm{GC}V_{E}\;$provides a useful measure of the relative additive genetic variation in residual variance, and, therefore, the potential response to selection. The moderate estimates observed in this study suggest that meaningful reductions in residual variance are achievable.

From a practical standpoint, genetic gain for residual variance is expected to be gradual, so selection for uniformity should be considered a long-term objective. Its effectiveness depends on factors such as the accuracy of estimated breeding values and the size of the reference population, both of which are directly influenced by data structure and recording practices. In practice, selection for reduced variability is implemented alongside selection for the mean using index-based approaches. Even modest gains in uniformity can accumulate over time, improving predictability and reducing management costs. Taken together, these results support the inclusion of residual variance in breeding objectives, particularly in large-scale commercial populations. Incorporating variability into selection decisions can reduce the frequency of extreme or suboptimal phenotypes, thereby improving production efficiency, management predictability, and animal welfare. More uniform animals may be less likely to require management interventions, which can contribute to reduced economic risk and improved robustness at the herd level, although the magnitude of these benefits may depend on the production system and management conditions.

From an implementation perspective, genetic heterogeneity of residual variance modeling involves several important methodological considerations. REML-based approaches, such as DHGLM, are relatively computationally efficient and can be integrated into existing genetic evaluation systems. However, these methods offer limited flexibility for modeling more complex or hierarchical structures. In contrast, Bayesian models are more computationally demanding but provide greater flexibility, allowing the incorporation of hierarchical relationships and the estimation of full posterior distributions. In our study, both approaches produced comparable estimates under similar data conditions. Ultimately, the choice between them should reflect a balance between computational resources, modeling flexibility, and the specific objectives of the breeding program.

Recently, Berghof et al. (2019b) presented an alternative way to define uniformity (or resilience) traits by measuring deviations from expected production levels over a period of time. In that study, several indicators for uniformity were presented, including variance of deviations, skewness of deviations, slope of reaction norms, and autocorrelation of deviations. Using simulation scenarios in pigs and dairy cattle, they showed that incorporating resilience-related indicators into selection indices, weighted by their economic relevance (eg reduced labor and health interventions), can improve overall selection response. Although these results were obtained under simulated conditions and in different species, they highlight the potential value of incorporating variability-related traits into breeding objectives. Therefore, while further empirical validation in beef cattle populations is needed, uniformity traits may represent a relevant component of future total merit indices.

Additionally, some of the challenges in selecting for uniformity stem from limitations in data structure and collection, which are directly relevant to the interpretation of the results obtained in this study. Unbalanced datasets, or those that do not capture the full range of management environments, can lead to underestimation of genetic variance for residual variance, low heritability, and artificially high genetic correlations between trait means and their residual variances. In the present study, the imposed minimum CG sizes may have contributed to these limitations. Therefore, data collection strategies should better reflect the complexity of real-world production systems. Because animals are raised under diverse environmental conditions, this variation must be adequately represented in the data. Balanced sampling across management types, combined with longitudinal data collection, is particularly important for minimizing confounding effects, accounting for genotype-by-environment interactions, and improving the reliability of variance estimates. Advancements in precision livestock farming technologies have further expanded the possibilities for monitoring traits associated with uniformity. High-throughput tools such as sensors, imaging systems, and wearables enable continuous, objective phenotyping across a range of production contexts (Morota et al. 2026), thereby supporting more accurate estimation of variability-related parameters. These technologies are already being used in other species to capture traits like locomotion, leg conformation, and feeding behavior (Morota et al. 2018; Psota et al. 2020), and they hold strong potential for adaptation to beef cattle systems.

The advent of genomic selection has improved selection responses in many livestock species (Meuwissen et al. 2001; Hayes et al. 2009; Goddard et al. 2010). However, to date, genomic approaches to quantify genetic heterogeneity of residual variance are scarce, likely because DHGLM is computationally demanding to fit high-dimensional genomic data. In this study, genomic data were not used because the model failed to converge. Nonetheless, the use of genomic selection to predict uniformity traits remains promising (Amorim et al. 2026). Integrating genomic data with time-series phenotypes and innovative uniformity indicators presents a promising direction for both research and practical applications. These approaches can enhance the understanding of genetic architecture underlying phenotypic variability and support the development of breeding programs aimed not only at improving performance but also at increasing uniformity and resilience across diverse production environments.

## Conclusion

This study estimated genetic parameters for residual variance in growth traits of American Angus cattle using heterogeneous variance models. Our results showed the presence of genetic variation not only for the mean of BW, WW, and YW, but also for their residual variance, although heritability estimates for residual variance were low. Estimates of$\;\mathrm{GC}V_{E}\;$indicated that selection to reduce variability is feasible. Genetic correlations between mean and residual variance differed across traits, with negative estimates for BW and positive estimates for WW and YW, suggesting trait-specific implications for selection. Differences between REML and Bayesian approaches were observed but were generally consistent in magnitude. These findings support the potential inclusion of variability-related parameters in genetic evaluation, while highlighting the need for further research to improve their estimation. Future research should explore the integration of genomic information into models of residual variance to improve prediction accuracy and support the inclusion of uniformity traits in genomic selection programs.

## Data Availability

The data supporting the results of this article are property of the American Angus Association cattle producers, and this information is commercially sensitive, therefore the data cannot be made available.

## Abbreviations

BW: birth weight
CG: contemporary groups
DHGLM: double hierarchical generalized linear model
REML: restricted maximum likelihood
WW: weaning weight
YW: yearling weight
